# Prediction of Protein Targets in Ovarian Cancer Using a Ru-Complex and Carbon Dot Drug Delivery Therapeutic Nanosystems: A Bioinformatics and µ-FTIR Spectroscopy Approach

**DOI:** 10.3390/pharmaceutics16080997

**Published:** 2024-07-27

**Authors:** Maja D. Nešić, Tanja Dučić, Branislava Gemović, Milan Senćanski, Manuel Algarra, Mara Gonçalves, Milutin Stepić, Iva A. Popović, Đorđe Kapuran, Marijana Petković

**Affiliations:** 1 Center for Light-Based Research and Technologies COHERENCE, Department of Atomic Physics, Vinča Institute of Nuclear Sciences, National Institute of the Republic of Serbia, University of Belgrade, 11000 Belgrade, Serbia; mstepic@vin.bg.ac.rs (M.S.); ivavukicevic@vin.bg.ac.rs (I.A.P.); djordje.kapuran@vin.bg.ac.rs (Đ.K.); marijanapetkovic@vin.bg.ac.rs (M.P.); 2 ALBA-CELLS Synchrotron, 08290 Cerdanyola del Vallès, Spain; 3 Laboratory for Bioinformatics and Computational Chemistry, Vinča Institute of Nuclear Sciences, National Institute of the Republic of Serbia, University of Belgrade, 11000 Belgrade, Serbia; gemovic@vin.bg.ac.rs (B.G.); sencanski@vin.bg.ac.rs (M.S.); 4 INAMAT2—Institute for Advanced Materials and Mathematics, Department of Science, Public University of Navarre, Campus de Arrosadia, 31006 Pamplona, Spain; manuel.algarra@unavarra.es; 5 CQM—Centro de Química da Madeira, Universidade da Madeira, 9020-105 Funchal, Portugal; maraisabel@staff.uma.pt

**Keywords:** nano-based anticancer drug delivery systems, protein targets, carbon dots, bioinformatics, µFTIR

## Abstract

We predicted the protein therapeutic targets specific to a Ru-based potential drug and its combination with pristine and N-doped carbon dot drug delivery systems, denoted as RuCN/CDs and RuCN/N-CDs. Synchrotron-based FTIR microspectroscopy (µFTIR) in addition to bioinformatics data on drug structures and protein sequences were applied to assess changes in the protein secondary structure of A2780 cancer cells. µFTIR revealed the moieties of the target proteins’ secondary structure changes only after the treatment with RuCN and RuCN/N-CDs. A higher content of α-helices and a lower content of β-sheets appeared in A2780 cells after RuCN treatment. Treatment with RuCN/N-CDs caused a substantial increase in parallel β-sheet numbers, random coil content, and tyrosine residue numbers. The results obtained suggest that the mitochondrion-related proteins NDUFA1 and NDUFB5 are affected by RuCN either via overexpression or stabilisation of helical structures. RuCN/N-CDs either induce overexpression of the β-sheet-rich protein NDUFS1 and affect its random coil structure or interact and stabilise its structure via hydrogen bonding between -NH2 groups from N-CDs with protein C=O groups and –OH groups of serine, threonine, and tyrosine residues. The N-CD nanocarrier tunes this drug’s action by directing it toward a specific protein target, changing this drug’s coordination ability and inducing changes in the protein’s secondary structures and function.

## 1. Introduction

Epithelial ovarian cancer accounts for 90% of all ovarian cancer diagnoses and is one of the most frequent and lethal types of gynaecological malignancies worldwide [1]. There are multiple reasons, including asymptomatic behaviour, diagnosis at advanced stages of the disease, recurrence, and resistance to chemotherapy, for this high mortality rate. Consequently, exploring new potential therapeutic targets related to overcoming drug resistance mechanisms and new treatment strategies is of great need for ovarian cancer patients. Nanotechnology is a part of the solution since the number of nano-based treatments and components for cancer-oriented theranostic techniques in clinical use has progressively increased [2]. Cancer nanomedicine predominantly focuses on improving pharmacokinetics and pharmacodynamics, targeting capability, and increasing localised drug efficacy [3]. Furthermore, enhanced therapeutic specificity can help eliminate the systemic toxicities associated with traditional therapies and improve prognosis and patient quality of life. Nanoformulations that fulfil all the tasks mentioned above, are nanocarriers (NCs) for anticancer drugs, which can act based on passive and/or active targeting [4]. For example, NCs can be designed to release drugs under external stimulation, such as illumination, magnetic field, and hyperthermia, a process known as active targeting. On the other hand, passive targeting and local drug activation are based upon specific cancer-sounding conditions such as pH, oxidative/reductive environments, and enhanced permeation and retention (EPR), wherein NCs can predominantly accumulate within tumour tissue [5]. Moreover, NCs can be designed to tune the mechanism of a drug’s action by interacting with specific biomolecule targets.

In the past few years, molecular targets and signalling pathways specific to cancer cells have been identified, enabling the development of new treatment strategies with advanced efficacy, selectivity, and reduced systemic toxicity. Along with nucleic acids, proteins have been recognised as drug targets for cancer therapeutics since they are essential in cancer cell growth, differentiation, metabolism, and survival. Proteins specific for cancer cells and those overexpressed in cancer cells stand out as potential drug targets. Some are membrane receptors, while others are markers on the cancer cell surface or overactive enzymes involved in specific signalling networks that promote or sustain cancer development [6,7,8,9,10]. Overexpressed membrane receptors or markers can serve as targets for drug docking and/or specific cell penetration of a drug.

On the other hand, overexpression of some specific enzymes, e.g., cyclin-dependent kinases (CDK4, CDK6, and CDK2), polo-like kinase 1 (PLK1), and aurora kinases (aurora A and aurora B), can induce or contribute to tumorigenesis, which drives cancer progression [10,11]. Targeting these signalling pathways and proteins responsible for cancer progression has become the primary strategy used in developing new drug therapies approved by multiple regulatory authorities (e.g., the European Medicines Agency (EMA),the U.S. Food and Drug Administration, the FDA). Therefore, choosing a suitable drug carrier could help tune drug properties to target key proteins. Although much experimental research has been conducted in the past decades, identifying drug–protein interactions remains tedious and time-consuming. Computational approaches have been developed to predict biological targets that can improve drug discovery success rates and simultaneously reduce experimental time and costs.

This study aims to explore the changes in the proteins of ovarian cancer cells caused by a potential drug Ru-complex (RuCN) and nanosystems made of NCs, carbon dots (CDs), and N-doped carbon dots (N-CDs)) and RuCN, denoted as RuCN/CDs and RuCN/N-CDs. A Ru complex with an η^5^-cyclopentadienyl ligand, [Ru(η^5^-C_5_H_5_)(PPh_3_)_2_CN], was selected according to its attractive photochemical and pharmacological properties for cancer therapy [12,13,14]. Recently, it was shown that ruthenium cyclopentadienyl complexes could act as protein kinase inhibitors and reactive compounds, which can induce the cleavage of protection groups in a biological environment [15,16,17]. CDs and N-CDs were used as NCs since they possess all the required properties: surfaces that can be easily modified and functionalised to interact with drug functional groups and biomolecules, high water dispersity, and good biocompatibility. This system showed promising potential for photodynamic therapy against CAL72 and A2780 cancer cell lines [18]. However, its mechanism of action without light activation has not been investigated in detail. Therefore, in this study, we used synergy between computational biology and FTIR spectroscopy to address the influence of RuCN with and without NCs on protein structure and function in the A2780 cancer cell line, which was used as a model system for human epithelial ovarian cancer.

In this study, we employed a computational evaluation of protein structures and an experimental approach involving the use of µFTIR to evaluate the effect of the analysed nanoparticles on total cellular protein status. We investigated the signalling pathways and protein–protein interaction (PPI) networks that are affected by the potential drug, RuCN, and nanotherapeutics based on RuCN and CDs or N-CD NCs (RuCN/CDs and RuCN/N-CDs), which are relevant to ovarian cancer. We aimed to assess the protein conformations and monitor any eventual conformational changes of secondary protein structures induced by the abovementioned treatments. To achieve this, we applied synchrotron-based Fourier transform infrared microspectroscopy (µFTIR), an effective and sensitive method for detecting proteins’ secondary structures and managing the overall protein conformational changes occurring in cells when exposed to treatments. Furthermore, based on recorded structural changes of proteins in A2780 cells resulting from nanosystems, we discussed the role of (N-)CD NCs’ surface chemistry and their effects on the complexity of the RuCN-mediated anticancer mechanism in the A2780 cancer cell type.

## 2. Materials and Methods

### 2.1. Materials

Organoruthenium complex, cyano(η^5^-cyclopentadienyl)bis(triphenylphosphine) ruthenium(II) [Ru(η^5^-Cp)(PPh_3_)_2_CN], denoted in the text as RuCN, was prepared and characterised according to established methods [13]. CDs and N-CDs were designed and characterised as previously described [19,20]. Nanocomposite systems made of RuCN and CDs or N-CDs (RuCN/(N-)CDs) were prepared by mixing RuCN with CDs or N-CDs over 24 h in a shaker at room temperature in the dark. Our previous publication described the structural arrangements of NPs and RuCN [18].

The A2780 human ovarian cancer cell line was obtained from the European Collection of Cell Cultures (ECACC, Salisbury, UK). The cell culture medium, foetal bovine serum (FBS), and antibiotics were purchased from Life Technologies (Paisley, UK).

### 2.2. Bioinformatics Analysis

The Informational Spectrum Method (ISM) was used to predict RuCN and (N-)CD protein targets in A2780 ovarian cancer cell line. ISM involves carrying out three major steps for prediction if two molecules are potential interactors: 1. Present molecules as a series of numbers based on Electron–Ion Interaction Potential (EIIP) [21]; in proteins, for example, each amino acid in the sequence of a protein’s primary structure is replaced by the value of EIIP for that amino acid. 2. Then, transform this numerical sequence into an Informational Spectrum (IS) using Fourier Transform. 3. Finally, multiply the spectra of two molecules to generate a cross-spectrum (CS). If the peaks in the ISs of two molecules are at the same frequency, this could indicate their long-term molecular interaction, especially if the most pronounced peak in the CS of these two molecules is at the same frequency. Therefore, we used CSs of system components, RuCN, CDs, and N-CDs, paired with each of the A2780 proteins to predict the possible physical interactions. Step 1 of this procedure for RuCN, CDs, and N-CDs was conducted using the Informational Spectrum Method applied for Small Molecules (ISM-SM) described in [22].

### 2.3. Preparation of A2780 Cells for µFTIR

A2780 cells were grown in RPMI-1640 medium containing 10% FBS and 1% antibiotic–antimycotic solution on a round 0.5 mm thick CaF_2_ carrier and incubated with 12.5 μM of RuCN containing 20 μg/mL of either CDs or N-CDs for 24 h, as described in our previous publication [18]. Untreated cells were used as control. The samples were prepared and stored lyophilized in a dry atmosphere until µFTIR spectra acquisition. Lyophylisation results in a minimum contribution of water-related signals in FTIR spectra.

### 2.4. Synchrotron Radiation Fourier Transform Infrared Micro-Spectroscopy (µFTIR)

The protein structures of A2780 cells before and after the treatment with RuCN, RuCN/CDs and RuCN/N-CDs were analysed using µFTIR (Synchrotron ALBA, MIRAS beamline, Barcelona, Spain). Synchrotron light was used as an IR source. A 3000 Hyperion microscope was coupled to a Vertex 70v spectrometer; mercury cadmium telluride (MCT) cooled with liquid nitrogen was used as a detector. The aperture of the FTIR microscope was set to the size of a single cell (12 × 12 µm^2^), and fifty cells were analysed from each group. Three independent replicates were conducted by co-adding 256 spectra at 4 cm^−1^ resolution. The spectroscopic data were collected in transmission mode using the 36 Schwarzschild objective and condenser. Spectra for each treatment were collected in the 4000–900 cm^−1^ mid-IR range.

### 2.5. Data Processing and Statistical Evaluation

The OPUS 8.2 (Bruker, Ettlingen, Germany) software was used for µFTIR data acquisition. Quasar software package Version 1.5.0. (Bioinformatics Laboratory of the University of Ljubljana, Slovenia) was employed to process protein region spectra, incorporating rubber band baseline correction and vector normalization for each spectrum. Second-derivative calculations were made by utilising a Savitzky–Golay filter with 15 smoothing points and a polynomial order of 3.

Statistical tests were performed with GraphPad Prism 7. Differences between means were considered significant when the *p*-value of the One-Way ANOVA test was less than 0.05.

## 3. Results and Discussion

### 3.1. Predicting Targets and Underlying Therapeutic Mechanisms of RuCN, RuCN/CDs, and RuCN/N-CDs in A2780 Ovarian Cancer Cells

Specific signalling networks are known to promote or sustain cancer, and exploring hot spots in proteomic networks represents a suitable approach to developing target-specific therapeutics. In this work, we explore protein candidates that could serve as targets for RuCN, RuCN/CDs, and RuCN/N-CDs by using the Informational Spectrum Model (ISM). As a potential pool of protein interactors with RuCN, RuCN/CDs, and RuCN/N-CDs, we used protein products of genes with baseline expression in A2780 cells. For this, we downloaded the list of genes from Expression Atlas with TPM ≥ 2.0, which was detected in the RNA-seq analysis of 934 human cancer cell lines (E-MTAB-2770) [23]. This list contained Ensemble identifiers of genes, but we needed the FASTA format of protein sequences for further research. We used the UniProt Retrieve/ID mapping tool to obtain 11 288 protein sequences that were further analysed as potential interactors with RuCN and CDs using the ISM [24].

**Figure 1:**
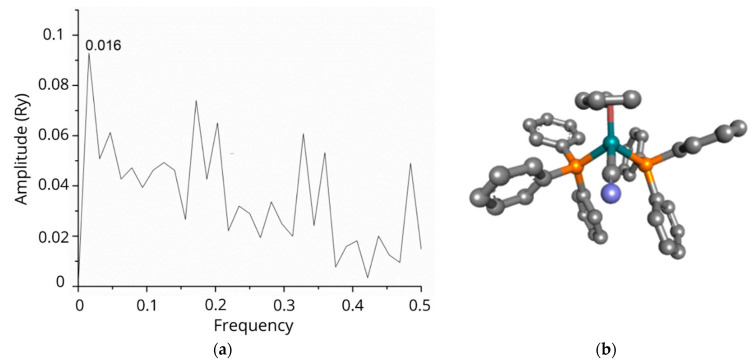
The informational spectrum of RuCN has a dominant peak at a frequency 0.016 (**a**). The molecular structure of the RuCN is given in (**b**), in which the cyan blue colour denotes the Ru-central metal ion, grey represent carbon-atoms, blue is the Cl, whereas the phosphorous atom is labelled with mustard colour.

ISM analysis showed that the tested N-CDs exhibited informational spectra identical to those of RuCN, with the dominant peak at a frequency of 0.016 (Figure 1a). Therefore, because the Ru-complex is a common part of both tested nanocomposite systems, we used RuCN as a model for cross-spectral analysis and paired it with each of the potential protein interactors in A2780 cells, assuming that the RuCN would target proteins in these cells [25,26].The ISM analysis resulted in a list of 595 proteins with a dominant peak in the CS with RuCN at a frequency of 0.016. Afterwards, we used these proteins in an enrichment analysis conducted using the DAVID (Database for Annotation, Visualisation, and Integrated Discovery) tool to determine which pathways/Gene Ontology (GO) terms were enriched in this list [27]. Functional annotation clustering revealed that mitochondrion-related proteins were the cluster with the highest enrichment score, namely, 4.96 (Table 1). This cluster encompasses proteins associated with keywords such as “respiratory chain”, with 14 proteins from our list (*p* = 2.6 × 10^−6^, after Benjamini correction), and “mitochondrion inner membrane”, with 19 proteins from our list (*p* = 2.0 × 10^−2^, after Benjamini correction). This cluster’s most abundant protein groups were NADH dehydrogenases and mitochondrial ribosomal proteins (Table 1). The cluster with chromatin-related proteins also had an enrichment score higher than 4.00 (Table S1) and encompasses proteins related to keywords such as “nucleosome core”, with 13 proteins from our list (*p* = 3.9 × 10^−4^, after Benjamini correction), and “chromosome”, with 26 proteins from our list (*p* = 8.6 × 10^−3^, after Benjamini correction). The most abundant proteins in this cluster are histones, as indicated in Table 1.

The most significant KEGG (Kyoto Encyclopedia of Genes and Genomes) pathway enriched in our protein list was the oxidative phosphorylation pathway (*p* = 5.1 × 10^−5^, after Benjamini correction), which included 18 proteins from the list. In addition, there were three terms from the Biological Process sub-ontology of Gene Ontology (GO) [28,29] that were significantly enriched in our protein list (with *p* ≤ 0.05 after Benjamini correction): (i) chromatin silencing (*p* = 8.4 × 10^−6^), with 13 proteins;(ii) mitochondrial electron transport, specifically NADH to ubiquinone (*p* = 1.2 × 10^−3^), with 11 proteins; and (iii) mitochondrial respiratory chain complex I assembly (*p* = 8.9 × 10^−3^), with 11 proteins from our list.

#### PPI Networks

In the next part of this work, we focus on the products of genes previously shown to be involved in ovarian cancerogenesis and annotated in the Human Phenotype Ontology (HPO) list with the term Ovarian neoplasm (HP:0100615) [30]. Sixty-one genes on the HPO list were available online [31]. Since proteins do not act alone in the cell but rather make connections with other proteins to perform their functions, we aimed to predict whether RuCN targets some of the proteins in the PPI networks of these 61 ovarian-related proteins from the HPO list. For this purpose, we used the String database of PPIs [32], with settings adjusted to retrieve only PPIs obtained from experiments (the active interaction source) and 100 interactors to be shown as the maximum number. The PPI network was searched for proteins whose interactors were predicted to be RuCN targets via the ISM. Three ovarian-related proteins had five or more proteins in their PPI networks identified as targets by ISM, AKT1, MDM2, and PRKN (Table 2). This result implies that the functions and pathways in which these proteins are involved are the most affected by RuCN.

Most of the ISM targets present in the PPI networks of the ovarian-neoplasm-related proteins were specific for one network, suggesting that the interaction of RuCN with these proteins has a specific effect on the cells. Nevertheless, the proteins SMAD3, TERF2IP, and YWHAZ were present in three and four PPI networks (Table 2), indicating that RuCN’s interaction with these proteins could affect several pathways within the cell. The ISM target SMAD3 is part of the PPI networks of AKT1, SMAD4, and TGFBR2. TERF2IP is a member of the PPI networks of BRCA1, MRE11, NBN, and RAD50. YWHAZ is an interactor with AKT1, FGFR2, and MDM2.

The most-affected PPI networks (AKT1, MDM2, and PRKN) by RuCN and RuCN/(N-)CDs drive central signalling cascades critical in tumorigenesis. These PPI networks, involving different types of proteins, are often deregulated in multiple malignancies and considered essential targets for developing selective inhibitors to suppress cancer growth and/or disease progression. The MDM2/p53 PPI inhibitors, RG7112 and RO5503781, were the first to enter clinical trials for patients with advanced malignancies [33]. The success of potent MDM2/p53 PPI inhibitors has significantly sped up studies seeking to target other PPIs with chemical agents acting as anticancer drugs. AKT1 is mainly involved in several critical cellular pathways, including regulating cell growth and division; therefore, it has been validated as a promising therapeutic target [34]. Similar to the other two networks, PRKN controls cell growth, metabolism, differentiation, and apoptosis [11].

### 3.2. µFTIR Spectroscopy Study of Alterations of Cancer Cell Proteins’ Secondary Structures

**Figure 2:**
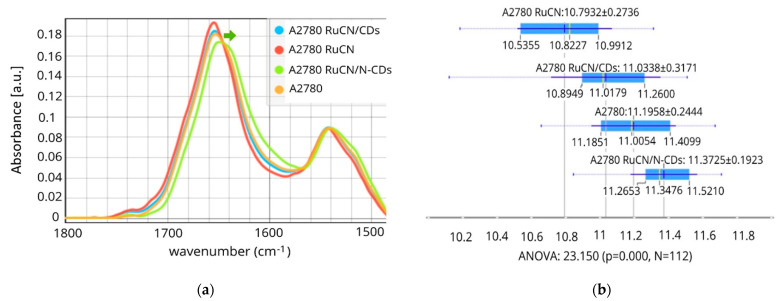
The µFTIR spectra of Amide I and Amide II regions, including the ester groups (1480–1800 cm^−1^). (**a**) Differences in the area of Amide I integration peaks (1600 and 1700 cm^−1^) (**b**). A green arrow indicates the downshift of the Amide I band after RuCN/N-CD treatment. (A2780 (control), N = 28; A2780 RuCN-treated cells, N = 28; A2780 treated with RuCN/CDs, N = 28; and A2780 treated with RuCN/N-CDs, N = 28). *p* < 0.05 represents a statistically significant difference.

After identifying potential RuCN protein targets and PPI networks (as discussed in the previous section), we focused on the proteins’ conformational changes in the cells and changes induced by RuCN without and with NCs (RuCN/CDs and RuCN/N-CDs). Interactions between drugs and proteins could alter secondary structures, folding, and protein functions. Consequently, protein structure changes provide insight into cancer cells’ responses to a drug. We aimed to investigate if adding NCs (CDs or N-CDs) tunes the mechanism of RuCN and whether the drug’s mode of action could be identified. To study changes in protein secondary structures, we applied FTIR, which generates a spectral signature of the macromolecular vibrations of a cell’s entire contents. This cell-specific signature is sensitive even to slight variations in chemical content and the 2D structures of proteins [35,36]. An infrared (IR) protein signature of the potential drug and its nanotherapeutic effects was defined as all the spectral variations in the protein fingerprint region of a cell due to a RuCN, RuCN/CDs, and RuCN/N-CDs exposure, respectively. Therefore, we recorded the IR spectra of untreated cancer cells and compared them to the spectra of the treated A2780 cells (Figure 2a).

Proteins exhibit nine characteristic groups of frequencies in their IR spectra. These groups arise from the polypeptide repeat units, distributed as follows: Amide A (~3300 cm^−1^), Amide B (3030–3100 cm^−1^), Amide I (~1650 cm^−1^), Amide II (~1550 cm^−1^), Amide III (1200–1400 cm^−1^), Amide IV (~735 cm^−1^), Amide V (~635 cm^−1^), Amide VI (~600 cm^−1^), and Amide VII (~200 cm^−1^) (Figure S1 and Table S2) [37,38]. Except for Amide I and II, amide bands are of little use in protein structural analysis due to their complexity and dependence on the nature of side chains and hydrogen bonding. Therefore, analyses of the spectral difference between untreated and treated cells are mainly based on the Amide I and II spectral band regions (1480–1700 cm^−1^) arising from C=O and C-N stretching vibrations, respectively, which will be addressed in this work.

Significant changes in spectra were observed after RuCN and RuCN/N-CDs treatment (Figure 2a), a result that was also confirmed by statistical analysis (Figure 2b), whereas RuCN/CDs did not affect cell protein structure. The most pronounced spectral differences compared to the control cells were observed as shifts in the position and intensity of the Amide I band and changes in the shape and intensity of the Amide II band after RuCN/N-CD treatment (Figure 2a). The Amide I and II band area was significantly reduced after the treatment with RuCN, while the RuCN/N-CDs positively affected the total protein content compared to control cells (Figure 2b), implying probable de novo synthesis of the proteins.

However, one can assume that an increase in the Amide I + Amide II area is caused by the superposition of protein- and N-CD-related signals. Namely, the FTIR spectra of CDs reveal a band at 1639 cm^−1^ caused by the presence of carbonyls (C=O) on the surface [19], while N-CDs have a band of a very strong intensity at 1647 cm^−1^ assigned to a C=O stretching vibration in primary and/or tertiary amides [39]. Both signals are in the region of the Amide I band of proteins. Our study observed a decreased intensity of the Amide I band of proteins (~1650 cm^−1^), as shown in Figure S1B, after treatment with N-CDs, whereas the treatment with CDs did not induce any changes in band intensity. If the observed changes were due to the superposition of signals from N-CDs or CDs, we would expect an increase in the intensity of the Amide I band because of the additive nature of overlapping peaks. Therefore, we can state that the decreased intensity of the Amide I band upon treatment with N-CDs indicates a deeper interaction mechanism between these NPs and proteins resulting in protein conformational changes.

Spectral shifting to lower wavenumbers was also observed, as the Amide I band of control cells (1651 cm^−1^) was shifted to 1648 cm^−1^ after the RuCN/N-CDs treatment. This band is associated with the random coil structure, which emphasises protein structure changes after the treatment [38]. This pronounced content of random coil structures present after the RuCN/N-CDs treatment was also confirmed via Principal Component Analysis (PCA) (Figure S2). Furthermore, it was demonstrated earlier that hydrogen bonding with the amide C=O groups results in a shift of the Amide I bands to lower wavenumbers [40], which indicates that RuCN/N-CDs interact with proteins via hydrogen bonding. Such a spectral downshift of Amide I was not detected after the treatment with structural analogue without an N-CD carrier (RuCN treatment), which indicates that amino groups present on the surfaces of N-CDs are involved in hydrogen bonding with the C=O groups from proteins. Even though RuCN did not induce such prominent spectral variations, the treatment of cells with this transition metal complex caused secondary structural changes in cell proteins, which were noticeable as a decrease in the Amide I band area (Figure 2b).

#### Changes in Individual Secondary Structures of Proteins

**Figure 3:**
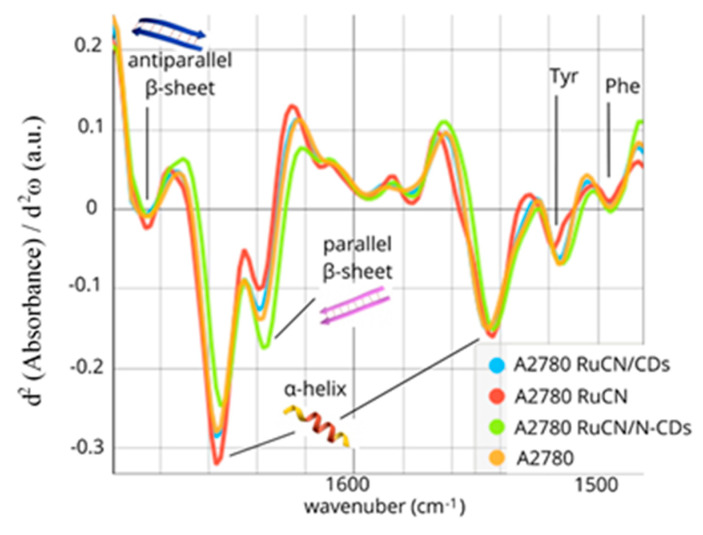
The second derivative of Amides I and II of the untreated cells (orange line) and cells treated with RuCN (red line), RuCN/CDs (blue line), and RuCN/N-CDs (green line).

The Amide I and II bands represent a mixture of secondary structure components characteristic of each cell type. Individual hidden absorption bands could be derived from the second-derivative spectra of Amides I and II. This approach allowed us to discern subtle changes in the secondary structures, thereby providing a more comprehensive understanding of the treatment effects. The second-derivative of the Amide I and II bands (1480–1700 cm^−1^), as shown in Figure 3, revealed that these bands consist of six sub-bands: the parallel β-sheet (1635 cm^−1^), the α-helix structure (1656 cm^–1^), turns and loops as well as antiparallel β-sheet structures (1685 cm^−1^), the α-helix (1543 cm^–1^), tyrosine (Tyr) (1515 cm^–1^), and phenylalanine (Phe) (1495 cm^−1^) side chains of amino acids. The identified bands were assigned to the Amide I and II region using previously described spectral components associated with different secondary structures [41,42,43,44,45] and given in Table 3.

The following changes were detected after the RuCN treatment: an increase in α-helix content and a decrease in parallel β-sheet content (Figure 3). On the contrary, a prominent increase in the number of parallel β-sheets and a reduction in the number of α-helices were observed when RuCN with an N-CD carrier was applied (the RuCN/N-CD treatment). Besides the quantitative decrease in the number of α-structures, their qualitative rearrangement was observed as a shift to the lower frequency (Figure 3). The interaction of cells with RuCN/N-CDs probably leads to a slight distortion in the α-helix structure or/and, partly, a α-helix–random coil transition. Changes in tertiary structure, resulting from changes in the relative orientations of some helical segments, can also be excluded. However, 3D structural changes in proteins after their interaction with carbon quantum dots have already been hypothesised [46], most likely resulting in changes in protein function. The treatment with RuCN/N-CDs also led to the following modifications in the Amide II region related to aromatic amino acid residues: (i) a significantly increased contribution of the band area at ≈1515 cm^−1^, i.e., of the “shoulder” peak associated with tyrosine (Tyr) indole ring vibrations (Figure 2a) [47], and (ii) a moderate increase in the band associated with phenylalanine (Phe) residue (Figure 3). In addition, the minimum position of PC2 at 1588 cm^−1^, which is related to the C—C stretching of phenyl rings, and the maximum at 1503 cm^−1^, which is associated with the C—H bending of phenyl rings [48,49], point to notable differences arising from the Phe residue as well (Figure S2). Moreover, an increase in the band at 1161 cm^−1^ that arise from the stretching vibrations of hydrogen-bonded C-OH groups of serine (Ser), threonine (Thr), and Tyr residues of cellular proteins was detected [50,51,52,53] (Figure S1B). Previously, the peaks at 1161 and 1172 cm^−1^ that originate mainly from the C-OH groups of Ser, Thr, and Tyr residues were observed in MCF-7 cells (from the human breast adenocarcinoma cell line) and colon tissues as well [53,54]. The importance of the -OH groups of these three amino acids is that they can be phosphorylated by several oncoproteins and, therefore, involved in essential signalling mechanisms that drive malignant cell characteristics [55]. As the cited authors indicated, the component bands at 1161 cm^−1^ arise from the stretching vibrations of hydrogen-bonded C-O groups, whereas the band at 1172 cm^−1^ is due to the stretching vibrations of non-hydrogen-bonded C- O groups. This result implies higher quantities of hydrogen-bonded Ser, Thr, and Tyr residues after RuCN/N-CDs treatment. These new hydrogen bonds could be formed between amino groups on N-CDs’ surface and -OH groups originating from Ser, Thr, and Tyr protein residues. The hydrogen bonding between amino groups from N-CD carriers and protein C=O groups was also ascribed to be the leading cause of the Amide I spectral downshift (Figure 2a).

### 3.3. Synergy of Bioinformatics and the FTIR Micro-Spectroscopy Approach

We used computational biology to identify specific protein targets for the anticancer drug RuCN and CD carriers for drug delivery and FTIR spectroscopy to provide a comprehensive understanding of their impact on cancer cell protein structure. In the next phase, we leveraged computational biology tools to elucidate structural changes’ underlying mechanisms and potential biological implications.

The most statistically significant change in the FTIR spectra after the RuCN/N-CD treatment was the parallel β-sheet increase (Figure 3 and Figure S2). This increase could be caused either by the stabilisation of β-sheet structures via protein interactions with RuCN/N-CDs (or treatment-induced “stabilisation factors”) or by the alterations in the expression levels and the de novo synthesis of proteins rich in the β-sheet structure after the treatment [56]. To reveal potential protein targets more precisely, we narrowed the number of possible mitochondrion-related proteins with the highest DAVID enrichment score to those with the highest content of the β-sheet structures (Table 1), and NDUFS1 stood out as the protein that could be overproduced after treatment or the most affected target protein for interaction with RuCN/N-CDs.

In the same way, the α-helix-rich proteins NDUFA1 and NDUFB5 stood out as potentially overexpressed proteins after RuCN treatment. An alternate option is stabilising helical structures in protein targets for RuCN through the interaction with the transition metal complex.

Furthermore, treating cells with RuCN/CDs did not induce any significant changes in the protein secondary structure, implying that Ru metallodrug interaction with CDs might sustain the coordination of RuCN with proteins.

We have shown that adding N-CDs to RuCN induces structural changes in proteins differently than the potential drug alone, implying there are different target molecules and therapeutic modalities. Even though the tested compounds had different protein targets (the suggested target proteins for RuCN are NDUFA1 and NDUFB5, and the target for RuCN/N-CDs is NDUFS1), the target proteins belong to the same protein groups, called NADH dehydrogenases. These enzymes play a crucial role in cellular respiration and energy production. Actively proliferating cancer cells require substantial amounts of NADH and NADPH. Therefore, selectively lowering NADH and NADPH levels is a promising cancer treatment strategy. Targeting these enzymes and their associated pathways has already been extensively studied as a potential therapeutic strategy for cancer treatment [57].

The effect observed in this work could be related to the results presented in our previous work [58], in which we demonstrated that coordination of the potential drug RuCN to an N-CD NC suppresses the level of oxidative stress while still maintaining the drug’s antitumor activity and potential to penetrate through the cell membrane. Therefore, it could be that combining this Ru drug with N-CD carriers balances the anticancer efficiency and leads to better therapeutical outcomes by reducing the oxidative stress linked to cancer progression, thus reducing the level of necrosis and inflammatory response. Additional therapeutical mechanisms might be the specific alterations in the protein secondary structure that the N-CDs in the nanocomposite system enable through specific interactions with the target NDUFS1 protein rich in β-sheet structures.

This combined approach enables the precise identification of protein targets for anticancer drugs through advanced computational models, facilitating tailored drug design.

## 4. Conclusions

Mitochondrion-related proteins were identified as potential targets for RuCN and RuCN/N-CDs. Interaction with the mentioned proteins might destabilise AKT1, MDM2, and PRKN signalling pathways, which control cancer cells’ survival, proliferation, invasion, apoptosis, and angiogenesis, making the combined drug’s therapeutic effect promising. We have demonstrated that combining RuCN with NCs may play a role in fine-tuning drug action and directing this drug toward a specific protein target through distinct interactions.

The results presented in this manuscript reveal that the treatment of ovarian cancer cells with RuCN induced a higher content of α-helices and a lower content of β-sheet structures compared to the untreated control and the overexpression of NDUFA1 and NDUFB5, respectively. On the other hand, coordination of the RuCN to the CD NCs blocked the coordination ability of the RuCN and did not affect the protein secondary structure of the A2780 cancer cell line. Furthermore, using N-CDs as NCs for the RuCN induced quite the opposite alterations in the secondary structures of ovarian cancer cellular proteins compared to those induced by the drug RuCN itself, which implies there are other protein targets for coordination and different therapeutic modalities. A substantially higher content of parallel β-sheets was observed, which highlighted β-sheet-rich protein NDUFS1 as the protein that could be overproduced after treatment or the most affected target protein for interaction with RuCN/N-CDs through the specific hydrogen bonding of the amino group from N-CDs with the protein C=O groups and -OH groups of Ser, Thr, and Tyr residues. Therefore, the correct choice of NC can provide new tools for further improvement, adaptation, and fine adjustment of drugs for specific protein targets in anticancer treatments. This could lead to rationally designed anticancer drug nanosystems targeting specific biomolecules or/and molecular events.

## Figures and Tables

**Table 1:** Cluster with highest DAVID enrichment score—mitochondrion-related proteins identified as potential targets of RuCN, with their secondary structures. Proteins coloured red are the ones with the highest percentages of α-helices, and those in blue are the ones with the highest percentages of β-sheets.

Protein Group	Protein	Part of the Protein with a Known Secondary Structure	Secondary Structure	Contribution of α-Helices and β-Sheets to the Protein Structure
NADH [ubiquinone] dehydrogenases	**NDUFA1**	1–70 (70 aa)	**5 helices** 11 helix–helix interactions, 5 beta turns, 1 gamma turn	**45.45%**
	NDUFA3	2–84 (84 aa)	**1 sheet** 1 strand **4 helices** 9 helix–helix interactions, 4 beta turns, 2 gamma turns	**8.30%** **33.33%**
	NDUFA8	4–172 (172 aa)	**1 sheet** 1 strand **7 helices** 14 helix–helix interactions, 17 beta turns, 4 gamma turns 1 disulphide	**3.22%** **22.58%**
	NDUFB1	3–58 (58 aa)	**4 helices** 10 helix–helix interactions, 6 beta turns	**40%**
	**NDUFB5**	52–189 (189 aa)	**6 helices** 20 helix–helix interactions, 5 beta turns, 2 gamma turns	**46.15%**
	NDUFB7	3–124 (137 aa)	**5 helices** 4 helix–helix interactions, 9 beta turns, 3 gamma turns, 1 disulphide	**29%**
	**NDUFS1**	30–716 (727 aa)	**6 sheets** 1 beta alpha beta unit, 6 beta hairpins, 3 beta bulges, 15 strands **27 helices** 33 helix–helix interactions, 82 beta turns, 14 gamma turns	**17.65%** **3.92%**
	NDUFS5	2–105 (106 aa)	**7 helices** 8 helix–helix interactions, 13 beta turns, 4 gamma turns, 2 disulphides	**26.92%**
	NDUFS6	29–123 (124 aa)	**2 sheets** 1 beta hairpin, 4 strands **2 helices** 1 helix–helix interaction, 11 beta turns,1 gamma turn	**9.52%** **9.52%**
Mitochondrial ribosomal proteins	MRPL18	1–180 (180 aa)	**2 sheets** 2 beta hairpins, 1 beta bulge, 5 strands **5 helices** 12 helix–helix interactions, 12 beta turns, 3 gamma turns	**6.66%** **16.66%**
	MRPL21	1–205 (205 aa)	**3 sheets** 3 beta hairpins, 5 beta bulges, 10 strands **1 helix** 1 helix–helix interaction, 11 beta turns, 1 gamma turn	**8.82%** **2.94%**
	MRPL36	1–103 (103 aa)	**1 sheet** 2 beta hairpins, 3 strands **1 helix** 6 beta turns	**7.69%** **7.69%**
	MRPL50	1–158 (158 aa)	**1 sheet** 1 strand **5 helices** 4 helix–helix interactions, 10 beta turns	**5.88%** **29.41%**
	MRPS30	1–439 (439 aa)	**2 sheets** 1 beta alpha beta unit, 3 beta hairpins, 3 beta bulges, 12 strands **12 helices** 14 helix–helix interactions, 29 beta turns, 10 gamma turns	**2.81%** **16.90%**
Mitochondrial ATP synthase subunits	ATP6V1F	1–119 (119 aa)	**1 sheet** 2 beta alpha beta units, 1 beta bulge, 3 strands **4 helices** 6 helix–helix interactions, 17 beta turns, 3 gamma turns	**6.89%** **13.79%**
NADH-ubiquinone oxidoreductases	MT-ND3	1–115 (115 aa)	**4 helices** 22 helix–helix interactions, 7 beta turns, 2 gamma turns	**30.70%**
	MT-ND6	1–174 (174 aa)	**9 helices** 30 helix–helix interactions, 12 beta turns, 6 gamma turns	**33.33%**
Cytochrome b-c1 complex subunits	UQCRH	17–91 (91 aa)	**4 helices** 7 helix–helix interactions, 5 beta turns, 1 gamma turn	**40%**
	UQCR1	2–63 (63 aa)	**3 helices** 6 helix–helix interactions, 6 beta turns	**33.33%**
HIG1 domain family member 1A, mitochondrial	HIGD1A	1–93 (93 aa)	**3 helices** 1 helix–helix interaction, 5 beta turns 7 gamma turns	**20%**
Dual specificity protein phosphatase 18	DUSP18	1–188 (188 aa)	**2 sheets** 2 beta hairpins, 1 beta bulge, 7 strands **8 helices** 8 helix–helix interactions, 8 beta turns, 4 gamma turns	**6.25%** **5.00%**
Cytochrome c oxidase subunit 7B, mitochondrial	COX7B	30–78 (80 aa)	**1 helix** 3 helix–helix interactions, 2 beta turns	**33.33%**

**Table 2:** ISM-identified potential protein targets in String PPI networks of proteins annotated with the term “Ovarian neoplasm” (HP:0100615) in HPO (HPO proteins).

HPO Protein	ISM Protein in the PPI Network of HPO Protein	S/N Value in the CS of ISM Protein and RuCN
MDM2 (6)	YWHAQ	11.68431
	RASSF3	9.9321
	**SFN**(AKT1)—2	8.26526
	UBE2E3	7.55273
	**ARRB1** (PRKN)—2	7.40497
	**YWHAZ** (AKT1, FGFR2)—3	6.60843
	SQSTM1	13.47355
PRKN (6)	LIMK1	11.41166
	PSMB4	10.03283
	RHOT2	9.66055
	CDK5	7.97013
	**ARRB1** (MDM2)—2	7.40497
	**SFN** (MDM2)—2	8.26526
	**HSPB1** (TP53)—2	8.24698
	**SMAD3** (SMAD4, TGFBR2)—3	8.13051
AKT1 (5)	EGLN1	7.58537
	**YWHAZ** (FGFR2, MDM2)—3	6.60843
	KDM6B	14.78335
SMAD4 (4)	**ACVR1B** (TGFBR2)—2	9.87143
	RAN	9.42564
	**SMAD3** (AKT1, TGFBR2)—3	8.13051
	ACACA	10.60752
BRCA1 (3)	**TERF2IP** (MRE11, NBN, RAD50)—4	9.37372
	FAM111A	6.8921
	BCL9	13.42896
CTNNB1 (3)	**CTNNA1** (CDH1)—2	10.23149
	PSEN2	8.155
	BAK1	16.43688
TP53 (3)	**HSPB1** (AKT1)—2	8.24698
	BCL2L2	7.12573
MRE11 (2)	**TERF2IP** (BRCA1, NBN, RAD50)—4	9.37372
	**CHAMP1** (RAD50)—2	9.33687
RAD50 (2)	**TERF2IP** (BRCA1, MRE11, NBN)—4	9.37372
	**CHAMP1** (MRE11)—2	9.33687
TGFBR2 (2)	**ACVR1B** (SMAD4)—2	9.87143
	**SMAD3** (AKT1, SMAD4)—3	8.13051
CDH1 (1)	**CTNNA1** (CTNNB1)—2	10.23149
CDKN2A (1)	PSMC3	7.13755
CHEK2 (1)	GINS2	6.01104
DICER1 (1)	TARBP2	6.7722
EWSR1 (1)	EIF4H	10.70126
FGFR2 (1)	**YWHAZ** (AKT1, MDM2)—3	6.60843
GATA4 (1)	FOS	11.15809
IDH1 (1)	DAZAP1	10.26606
LMNA (1)	LMNB2	11.67122
MSH2 (1)	FBXO5	8.00492
NBN (1)	**TERF2IP** (BRCA1, MRE11, RAD50)—4	9.37372
PALB2 (1)	MORF4L2	8.04956
RAD51 (1)	RAD51AP1	14.51291
RAD51D (1)	AMOTL2	7.63458

**Table 3:** Correlation between Amide I and II peak frequency positions obtained from the secondary-derivative spectrum and secondary structure assignments.

Frequency (cm^−1^)	Band Assignment
1685	Antiparallel β-sheet
1656	α-helix
1635	Parallel β-sheet
1543	α-helix
1515	Tyrosine (Tyr)
1495	Phenylalanine (Phe)

## Data Availability

Data will be made available at a reasonable request.

## Supplementary Materials

The following supporting information can be downloaded at: https://www.mdpi.com/article/10.3390/pharmaceutics16080997/s1, Figure S1: SR-FTIR spectra of Amide A, Amide B, (A) Amide I, II, and III regions (B) characteristic for protein analysis; Figure S2: PCA scores plot (A) and corresponding PCA loadings profile (B) of the second derivative data of the spectral region (1500–1700 cm^−1^) of control (orange) untreated cells, and cells treated with RuCN (red), RuCN/N-CDs (green) and RuCN/CDs (blue); Table S1: DAVID enrichment analysis on proteins identified as potential targets of RuCN; clusters with the highest Enrichment scores are shown; Table S2: Assignments of peak positions.
