# Supplementary material for: Prediction of Protein Targets in Ovarian Cancer Using a Ru-Complex and Carbon Dot Drug Delivery Therapeutic Nanosystems: A Bioinformatics and µ-FTIR Spectroscopy Approach
Source: Pharmaceutics. 2024 Jul 27;16(8):997. doi: 10.3390/pharmaceutics16080997 (PMC11359177; doi:10.3390/pharmaceutics16080997)
Supplement: Supplementary file 1 [file pharmaceutics-16-00997-s001.zip › pharmaceutics-3075942-supplementary.pdf]

Supplementary info

Prediction of protein targets in ovarian cancer using Ru-complex and carbon dots drug delivery therapeutic nanosystem: Bioinformatics and µ-FTIR spectroscopy approach

Maja D Nešić , Tanja Dučić , Branislava Gemović , Milan Senćanski , Manuel Algarra, Mara Gonçalves, Milutin Stepić, Iva Popović, Đorđe Kapuran and Marijana Petković

Cluster	Enrichment score	Proteins
**mitochondrion-related proteins**	**4.96**	NADH [ubiquinone] dehydrogenases: NDUFA1, NDUFA3, NDUFA8, NDUFB1, NDUFB5, NDUFB7, NDUFS1, NDUFS5, NDUFS6<br>Mitochondrial ribosomal proteins: MRPL18, MRPL21, MRPL36, MRPL50, MRPS14, MRPS30, MRPS33, MRPS35<br>Mitochondrial ATP synthase subunits: ATP5E, ATP5G2, ATP6V1F<br>NADH-ubiquinone oxidoreductases: MT-ND3, MT-ND6<br>Mitochondrial solute carriers: SLC25A19, SLC25A20<br>Cytochrome b-c1 complex subunits: UQCC3, UQCRH, UQCR10<br>Inorganic pyrophosphatase 2, mitochondrial – PPA2<br>HIG1 domain family member 1A, mitochondrial - HIGD1A<br>Dual specificity protein phosphatase 18 - DUSP18<br>Cytochrome c oxidase subunit 7B, mitochondrial - COX7B<br>Coiled-coil-helix-coiled-coil-helix domain-containing protein 1 - CHCHD1<br>Cardiolipin synthase (CMP-forming) – CRLS1<br>Acyl-CoA 6-desaturase – FADS2
**chromatin-related proteins**	**4.87**	Histones: H2A family: H2AC6, H2AC7, H2AC8, H2AC12, H2AC13, H2AC14, H2AC20, H2AC21, H2AFJ, H2AFX, H2AW; H3 family: H3C1, H3C14<br>Centromere proteins: CENPB, CENPQ<br>ZW10 interactor - ZWINT<br>Transcription factor jun-B - JUNB<br>Telomeric repeat-binding factor 2-interacting protein 1 – TERF2IP<br>Telomeric repeat-binding factor 1 – TERF1<br>Telomerase reverse transcriptase - TERT<br>Spindle and kinetochore-associated protein 3 – SKA3<br>RAD51-associated protein 1 – RAD51AP1<br>Protein timeless homolog - TIMELESS<br>Protein Mis18-beta – OIP5<br>Nuclear ubiquitous casein and cyclin-dependent kinase substrate 1 – NUCKS1<br>Mothers against decapentaplegic homolog 3 – SMAD3<br>Mortality factor 4-like protein 2 – MORF4L2<br>Mitotic checkpoint serine/threonine-protein kinase BUB1 – BUB1

	Methyl-CpG-binding protein 2 – MECP2
	High mobility group protein HMG-I/HMG-Y – HMGA1
	F-box only protein 11 – FBXO11
	DNA polymerase epsilon subunit 3 – POLE3
	COP9 signalosome complex subunit 9 – COPS9
	Chromosome alignment-maintaining phosphoprotein 1 – CHAMP1
	Barrier-to-autointegration factor – BANF1

Table S1. DAVID enrichment analysis on proteins identified as potential targets of RuCN; clusters with the highest Enrichment scores are shown.

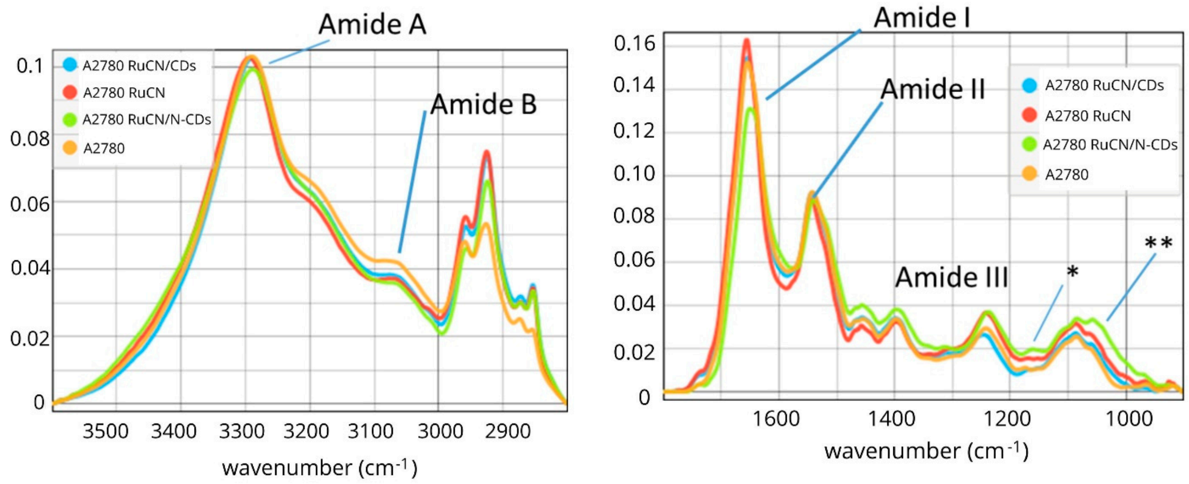

(**A**) (**B**)

Figure S1. SR-FTIR spectra of Amide A, Amide B, (**A**) Amide I, II, and III regions (**B**) characteristic for protein analysis. Increase in bands at 1161 (*) and 1055cm-1 (**) after the treatment with RuCN/N-CDs arise from stretching vibrations of hydrogen-bonded C-OH groups of Ser, Thr, and Tyr residues and C-OH stretching vibrations in carbohydrates, respectively.

IR peaks of peptidice bond. Assignments of peak positions Frequency/wavelength	Vibration type	Name	Note
3300 cm-^{1}/3 µm	N-H stretching	Amide A	Insensitive to backbone conformation<br>Sensitive to hydrogen bonding
3030–3100 cm^{-1}/3.3–3.2 µm	N-H Fermi resonance	Amide B	The resonance between amide A and an amide-II overtone
1650 cm^{-1}/6 µm	C=O stretching (with contribution from N-H and C-N deformation)	Amide I	Very sensitive to backbone conformation
1550 cm^{-1}/6.5 µm	N-H bending (with contribution from C-N, C-C and C-O)	Amide II	Correlation to backbone conformation is less established
1200–1400 cm^{-1}/8.5–7 µm	N-H bending, C-N stretching	Amide III	Weakly active, sensitive to the side-chain composition

Table S2. Assignments of peak positions

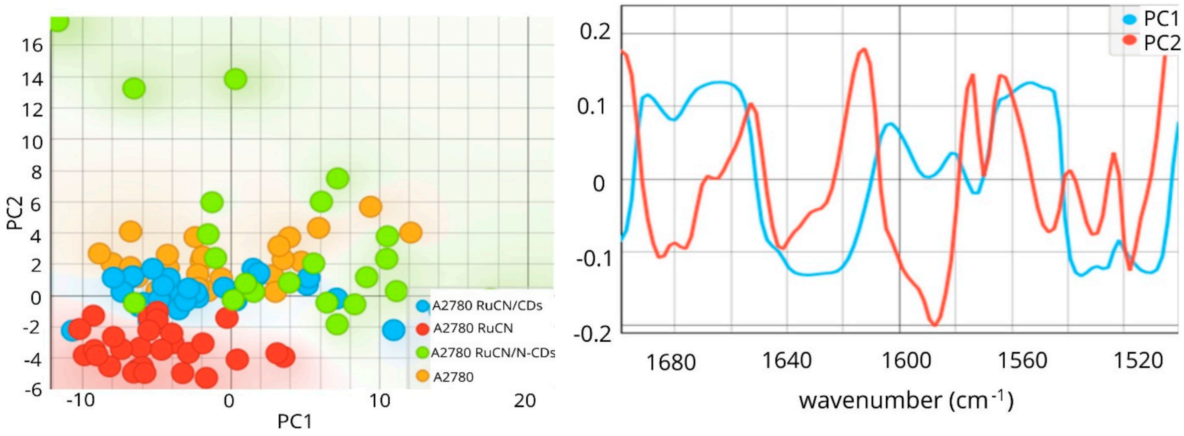

(**A**) (**B**)

Figure **S2.** PCA scores plot (**A**) and corresponding PCA loadings profile (**B**) of the second derivative data of the spectral region (1500 - 1700 cm^{-1}) of control (orange) untreated cells, and cells treated with RuCN (red), RuCN/N-CDs (green) and RuCN/CDs (blue). The PCA scores plot (**B**) denotes the variability associated with the first two components.

Amide I and II bands were investigated by PCA. PCA results of the FTIR spectral range (1480–1800 cm^{-1}) are presented as scores (Figure S2A) and loading plots (Figure S2B). The score plot shows the distribution of treated and untreated cells in the PC space and they revealed area separation and clustering of untreated A2780 cells (orange region) from RuCN (red region) and RuCN/NCDs (green region) treated. Further, the spectrum bands that most influence the clustering are examined through the PCA loading plots that identify the peaks (maximums and minimums of the spectra) which strongly affect the principal components. The PC1 showed broad peaks at ~1635 (minimum) and 1670 cm^{-1} (maximum), corresponding to lower content of the β-sheets structure and higher concentration of the turns and loops in the RuCN/N-CDs treated cells (Figure S2B). The second component of the PCA, PC2 showed the most prominent contribution of the band at 1615 and 1648 cm^{-1} (maximums) in the Amide I region. These peaks are associated with side chain structures and random coil organization in protein, respectively, pronounced mostly in control cells and RuCN/N-CDs (note that the PCA was performed on the second derivative data presented in Fig 3.). On the other hand, minimums (1642 and 1687 cm^{-1}) are related to β-sheets and turns and loops in the Amide I region, most pronounced in the RuCN-treated cells The minimum position of PC2 at 1588 cm^{-1} related to C—C stretching of phenyl rings and the maximum at 1503 cm^{-1} related to C—H bending of phenyl rings pointed to differences in the Amide II region associated with Tyr residue after cell treatment. In general, the analysis shows that there are clear differences in protein secondary contribution between untreated and RuCN (red region) and RuCN/NCDs (green) treated compared to untreated cells.
